# Evaluation of PCR conditions for characterizing bacterial communities with full-length 16S rRNA genes using a portable nanopore sequencer

**DOI:** 10.1038/s41598-020-69450-9

**Published:** 2020-07-28

**Authors:** So Fujiyoshi, Ai Muto-Fujita, Fumito Maruyama

**Affiliations:** 1 Office of Industry-Academia-Government and Community Collaboration, Hiroshima University, 1-3-2 Kagamiyama, Higashi-Hiroshima, Hiroshima 739-8511 Japan; 2 Laboratorio de Ecología Microbiana Aplicada, Departamento de Ciencias Químicas y Recursos Naturales, Scientific and Biotechnological Bioresource Nucleus (BIOREN-UFRO), Universidad de La Frontera, Ave. Francisco Salazar 01145, Temuco, Chile; 3 Data Science Center, Nara Institute of Science and Technology, Ikoma, Nara Japan

**Keywords:** Microbial ecology, Microbiome

## Abstract

MinION (Oxford Nanopore Technologies), a portable nanopore sequencer, was introduced in 2014 as a new DNA sequencing technology. MinION is now widely used because of its low initial start-up costs relative to existing DNA sequencers, good portability, easy-handling, real-time analysis and long-read output. However, differences in the experimental conditions used for 16S rRNA-based PCR can bias bacterial community assessments in samples. Therefore, basic knowledge about reliable experimental conditions is needed to ensure the appropriate use of this technology. Our study concerns the reliability of techniques for obtaining accurate and quantitative full-length 16S rRNA amplicon sequencing data for bacterial community structure assessment using MinION. We compared five PCR conditions using three independent mock microbial community standard DNAs and established appropriate, standardized, better PCR conditions among the trials. We then sequenced two mock communities and six environmental samples using Illumina MiSeq for comparison. Modifying the PCR conditions improved the sequencing quality; the optimized conditions were 35 cycles of 95 °C for 1 min, 60 °C for 1 min and 68 °C for 3 min. Our results provide important information for researchers to determine bacterial community using MinION accurately.

## Introduction

Most microbes in the natural environment have not yet been cultured, but recent molecular technological advances make it possible to study them without cultivation. New technologies have allowed breakthroughs to be made in the elucidation of roles of microbes in the natural environment and in the fields of human health; for example, in investigations of the human gut microbiome^1,2^ and in bio-engineering for agriculture, bioremediation and industry^3^. Molecular techniques have provided researchers with various analytical procedures for understanding microbial communities using clone libraries^4,5^, T-RFLP (terminal restriction fragment length polymorphism) analysis^6,7^, and DGGE (denaturing gradient gel electrophoresis) techniques^8,9^. Full-length bacterial 16S rRNA genes have historically been sequenced using conventional molecular cloning and Sanger sequencing, but this approach is time-consuming, expensive, and has low throughput^10^. Currently, MiSeq sequencing (Illumina, San Diego, CA, USA) is the most widely used platform for 16S rRNA gene amplicon sequencing for microbial community analysis. PCR is conducted on the variable regions (V2, V3, and V4) in bacteria, with the primers focusing on the conserved region of 16S rRNA^11^. MiSeq, which has become popular for its high precision (99.9%), enables the PCR amplicon sequence determination by merging overlapped region of paired 300 nt facing reads^12^. However, in terms of taxonomic resolution, comparative analyses have revealed the importance of the target region and the choice of the primer pair, as revealed by the following studies in this area. Cai et al.^13^ reported the effects of the 16S rRNA gene primer sets and recommended the use of V3 and V4 primer pairs for several environmental sample types. On the other hand, Guo et al*.*^14^ proposed the use of the V1 and V2 regions for analysing the functional bacterial groups in a sludge sample. However, Wang et al*.*^15^ recommended using the V5, V6, and V7 regions for ascertaining the bacterial community structure in aging flue-cured tobaccos because chloroplast and mitochondrial genes have lower co-amplification levels. Kindworth et al*.*^16^ showed that, based on the comparison of microbial community obtained using the multiple universal primer sets, each universal primer set generate significant differences in taxonomic spectrum. The short-read lengths (100–300-bp) inherent in the single universal primer set techniques also prevent species-level analyses in microbial ecology^17^.

In 2012, introduction of the high-throughput Pacific Biosciences (PacBio, Menlo Park, CA, USA) sequencer facilitated structural analysis of microbial communities^18^. The PacBio platform can obtain full-length 16S rRNA gene sequences, which increases taxonomic resolution by sequencing the number of the informative sites. Its primary limitations lie in its lack of versatility and exemplified by tedious sample preparation^19^. In 2014, the Nanopore MinION sequencer (Oxford Nanopore Technologies, London, UK), now regarded as breakthrough in DNA sequencing, was developed. It contains several intriguing features that enable real-time, on-site analyses of any genetic material. The device has been used in diverse ways in various fields, including drug-resistance gene analyses and assessment of the rapid gain in reptile and amphibian biodiversity in rainforests^20,21^. MinION starts to be used more often and its sequencing quality has been improving with higher sequencing read accuracy in 1D sequencing (94%). Recently, an increasing number of studies have reported their concerns about on-site and real-time measurements using MinION^22–24^. These MinION-based gene sequencing techniques have provided new insight into microbial community structures much more rapidly and easily than ever before. The optimization, establishment and standardization of methods for the quantitative evaluation of microbial composition in the environment are inevitable. This would allow scientists to accurately answer the fundamental question of microbial ecology: what kind of and how many microorganisms are present in the environment.

PCR-based 16S rRNA analysis of bacterial community structure is subject to biases from the PCR-related conditions. These include the template concentration, DNA polymerase choice, number of cycles used, amplification reaction time, and the reaction temperature^25–29^. Bacterial communities can also be reconstructed by only collecting the 16S rRNA sequences obtained from metagenomes, thereby avoiding PCR bias; however, PCR-free libraries require relatively large amounts of input DNA, and are impractical for many sample types^30^. Therefore, cost-effective marker gene amplicon sequencing is often preferred over metagenomic sequencing for microbial community analysis because it enables the assessment of uncultivable organisms.

With this background, the aim of this study was to evaluate MinION PCR conditions through three approaches: (1) sequencing the full-length bacterial 16S rRNA gene from a single bacterial species to examine our bioinformatics pipeline; (2) sequencing the amplicon of full-length bacterial 16S rRNA gene from three different types of bacterial mock community DNAs under five different PCR conditions; and (3) sequencing the amplicon of full-length 16S rRNA genes from six environmental samples to compare the results with those of bacterial 16S rRNA V3–V4 regions sequenced using MiSeq.

## Results

### MinION data filtering by length

**Figure 1:**
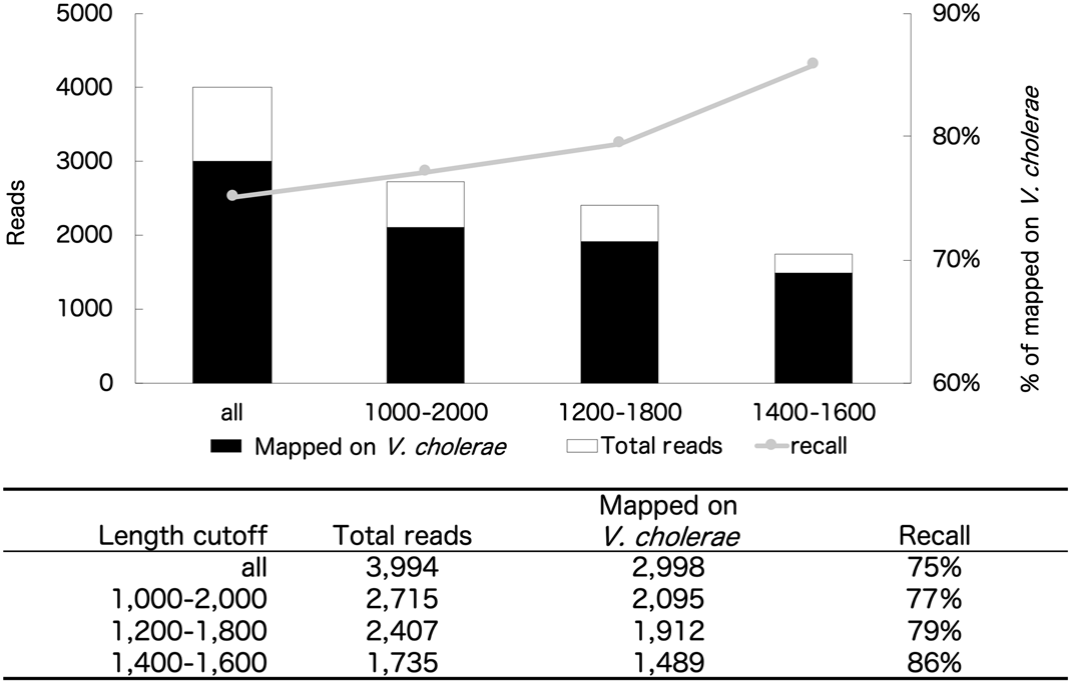
The effect of length trimming on *Vibrio cholerae* data.

We initially used the Ribosomal Database Project (RDP) classifier version 2.11 (https://rdp.cme.msu.edu/)^31^, and the RDP classifier 16S training set No:16 as database (https://sourceforge.net/projects/rdp-classifier/files/RDP_Classifier_TrainingData/RDPClassifier_16S_trainsetNo16_rawtrainingdata.zip/download). However, this tool erroneously assigned *Vibrio* as *Allomonas.* Analysis with another tool (mothur^32^) required an excessively long run time. We eventually chose to use Burrows-Wheeler Aligner (BWA-MEM, v. 15. 0.7 or v. 0.7. 17)^33^ with a database derived from the RDP^34^ as described in Methods. The MinION sequence length distribution and species identification accuracy were investigated using a single bacterial species, *Vibrio cholerae*. A commercially available kit (16S Rapid Sequencing Kit and 16S Barcoding Kit; Oxford Nanopore Technologies) with primers for full-length 16S rRNA amplicon sequencing on the MinION platform was used. The distribution of sequencing read lengths showed the highest frequency at around 1,500-base reads. Both shorter (5-base) and longer (200,000-base) reads also appeared. Three-step filtering ranging from 1,000–2,000 bases, 1,200–1,800 bases, and 1,400–1,600 bases was used to include the highest frequency length (1,500-base) in each step. As shown in Fig. 1, the hit ratio (*V. cholerae*/total reads) increased from 75% (2,998/3,994 reads) without filtering to 86% (1,489/1,735 reads) after filtering with 1,400–1,600 bases. Hence, we filtered the reads by length, using those in the 1,400–1,600 base range thereafter.

### PCR conditions based on mock communities

**Figure 2:**
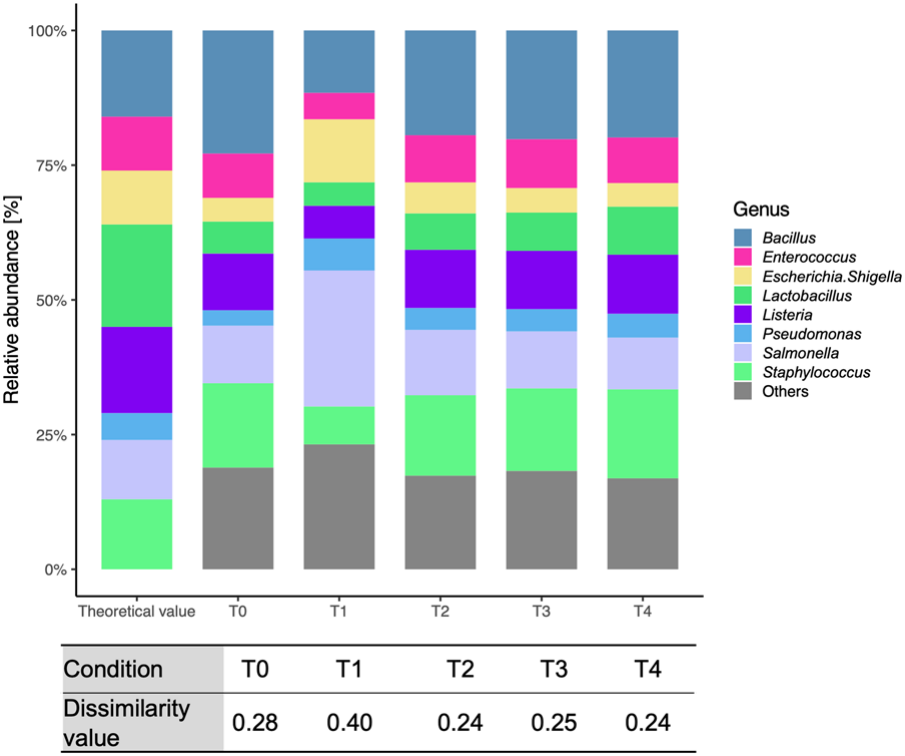
Comparison of five PCR conditions using a mock community. The theoretical value was evaluated by shot-gun sequencing using the Illumina Miseq (2 × 150 bp).

**Figure 3:**
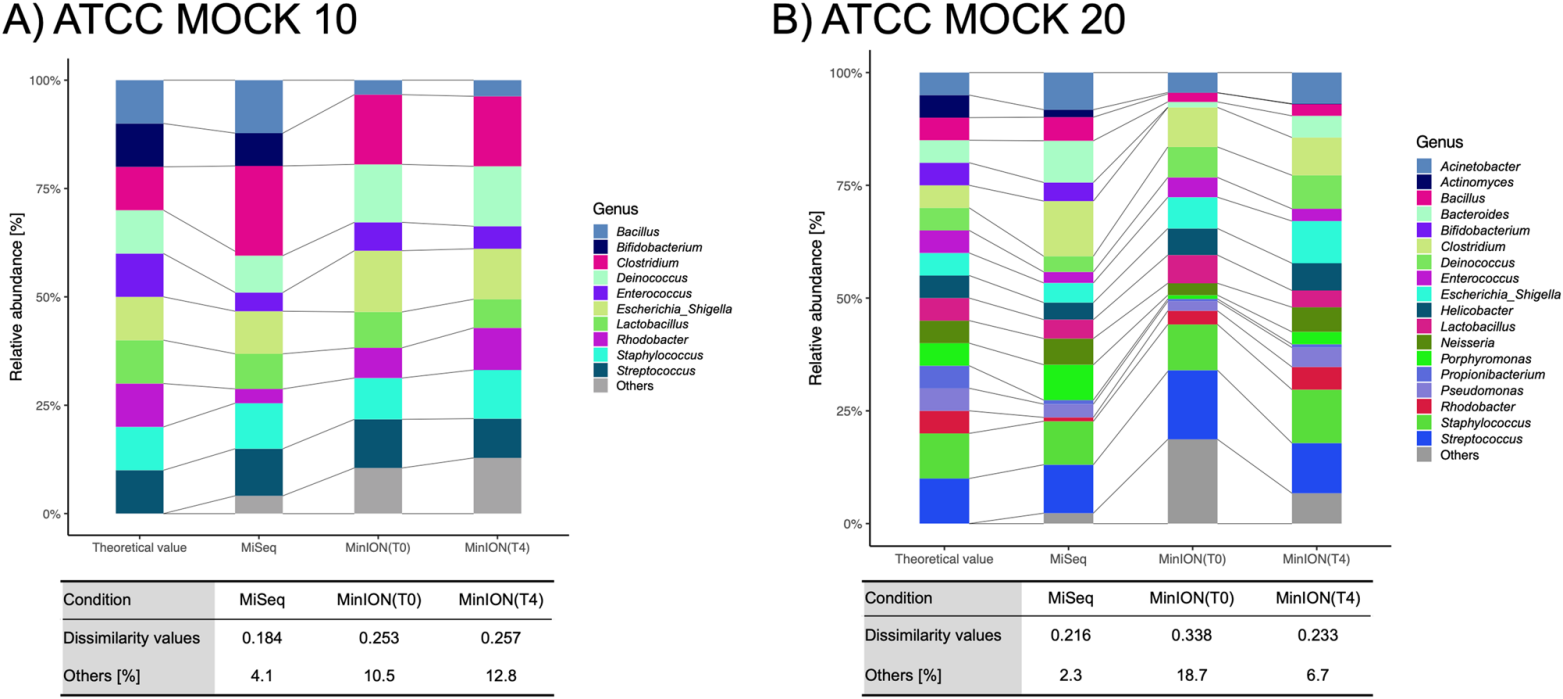
Comparison of bacterial community composition of mock 10 and 20 samples using MinION under the T0 and T4 conditions versus MiSeq. The theoretical values of mock ATCC 10 and 20 were evaluated by whole genome shot-gun sequencing using the Illumina platform (https://www.atcc.org/~/media/PDFs/Presentations/2017/Microbiome%20Reference%20Standards.ashx).

The results obtained from the ZymoBIOMICS mock community were compared for the five PCR conditions shown in Fig. 2, with detailed information for each condition being described in Table 1. The Goods coverage values were greater than 99% for all samples (Table 2). Bray–Curtis dissimilarity^35^ was used as a measure for assessing the difference between the observed and theoretical communities structures for each PCR condition. Bray–Curtis dissimilarity is bounded between 0 and 1, where 0 means that the two compared samples have the same composition, and 1 means the two sites do not share any species^36^. The dissimilarity value for PCR condition T0 was 0.28, whereas those for T1, T2, T3 and T4 were 0.40, 0.24, 0.25 and 0.24, respectively (in detail and species level data, see Supplemental Table S2). The initial T0 and prefered T4 conditions were also compared using two other mock communities (that is, an even mix of 10 strains; ATCC 10, and an even mix of 20 strains; ATCC 20) (Fig. 3). For ATCC 10, the dissimilarity values between the observed and theoretical values for T0 and T4 were almost the same, at 0.253 and 0.257, respectively. However, in the case of ATCC 20, the dissimilarity values for T0 and T4 were 0.338 and 0.233, respectively. This tendency was more pronounced at the species-level data (Supplemental Table S3). We obtained a bacterial community composition similar to the theoretical one under T4 conditions, compared with that obtained under the T0 conditions. Almost all the genera except for *Bifidobacterium* were detected under both conditions. When the ATCC 10 and ATCC 20 mock communities were analysed using MiSeq, the dissimilarity values were 0.184 and 0.216, respectively. These values are smaller than those for MinION under T0 and T4 conditions.

**Table 1:** PCR conditions used in this study.

**PCR condition Trial 0 (T0)**		
98 °C (Preheating)	2 min	35 cycles
98 °C (Denaturation)	10 s	
60 °C (Annealing)	15 s	
68 °C (Extension)	2 min	
**PCR condition Trial 1 (T1)**		
98 °C (Preheating)	2 min	35 cycles
98 °C (Denaturation)	10 s	
60 °C (Annealing)	15 s	
68 °C (Extension)	***3 min***	
**PCR condition Trial 2 (T2)**		
98 °C (Preheating)	2 min	35 cycles
98 °C (Denaturation)	10 s	
60 °C (Annealing)	***1 min***	
68 °C (Extension)	2 min	
**PCR condition Trial 3 (T3)**		
98 °C (Preheating)	2 min	35 cycles
98 °C (Denaturation)	10 s	
60 °C (Annealing)	***1 min***	
68 °C (Extension)	***3 min***	
**PCR condition Trial 4 (T4)**		
98 °C (Preheating)	2 min	35 cycles
95 °C (Denaturation)	***1 min***	
60 °C (Annealing)	***1 min***	
68 °C (Extension)	***3 min***	

Conditions that differ from T0 are shown in ***bold italic style***.

**Table 2:** Sequence reads and the proportions of “Unassigned” generated per sample.

Sample	Experiment condition	Base called reads	Quality filtered reads	Goods coverage	The proportions of “Unassigned” (%)
ZymoBIOMICS	MinION (T0)	172,907	127,931	99.7	0.2
	MinION (T1)	122,858	95,121	99.8	0.3
	MinION (T2)	403,620	338,055	99.6	0.3
	MinION (T3)	124,534	103,881	99.7	0.3
	MinION (T4)	188,203	153,855	99.6	0.3
ATCC10	MiSeq	168,892	–	99.3	0.0
	MinION (T0)	52,999	39,317	99.5	1.0
	MinION (T4)	29,652	23,404	99.8	0.0
ATCC20	MiSeq	145,256	–	99.1	0.0
	MinION (T0)	41,745	12,838	99.5	9.7
	MinION (T4)	22,199	12,370	99.8	0.0
B2	MiSeq	312,725	–	99.1	21.8
	MinION (T0)	158,735	50,615	99.4	2.1
	MinION (T4)	14,607	6,896	99.4	0.0
B6	MiSeq	222,258	–	99.5	0.1
	MinION (T0)	110,380	24,681	99.1	12.0
	MinION (T4)	12,176	6,003	99.1	0.0
B7	MiSeq	219,844	–	97.0	0.1
	MinION (T0)	194,442	35,342	96.5	15.1
	MinION (T4)	63,622	52,952	96.8	0.0
B11	MiSeq	300,949	–	98.5	18.7
	MinION (T0)	55,562	28,028	98.5	1.6
	MinION (T4)	29,242	21,861	98.8	0.0
B13	MiSeq	410,523	–	96.3	4.2
	MinION (T0)	108,020	34,912	97.0	1.5
	MinION (T4)	53,586	45,919	96.8	0.0
B14	MiSeq	226,434	–	98.6	26.2
	MinION (T0)	229,720	144,507	99.1	0.6
	MinION (T4)	81,806	61,098	98.6	0.0

### PCR conditions using environmental samples and MiSeq sequencing

**Figure 4:**
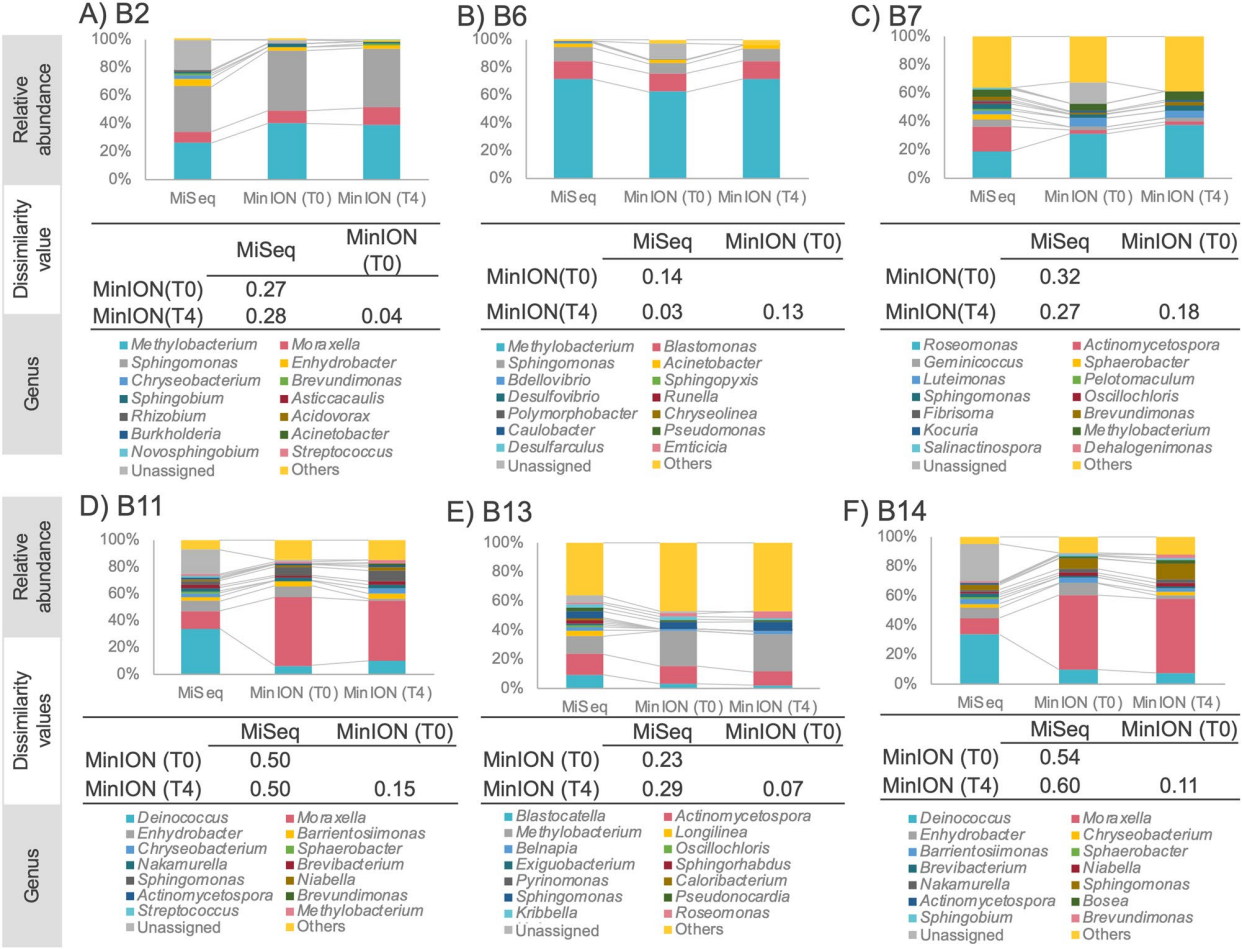
Comparison of the bacterial community composition of environmentally-sourced samples using MinION under T0 and T4conditions versus MiSeq. The top 15 most abundant genera are listed.

We applied our optimized PCR condition (T4) to environmental samples comprising bathtub inlet biofilms, showerhead feed water and showerhead biofilms from a bathroom (n = 6 samples). Each extracted DNA prepared from these samples was used separately as a PCR template, and 16S rRNA gene amplicon libraries were sequenced on both MinION and MiSeq platforms. The Goods coverage values were greater than 96% for all samples (Table 2). Figure 4 shows the 15 most prevalent genera in the samples. Under MinION T4 conditions, the genus distribution was similar to those under MiSeq, but only for the B6 sample (Fig. 4B). The remaining five samples from MinION with T4 output data resemble those generated under MinION T0 conditions. Table 2 lists the read numbers from MiSeq, MinION T0 and MinION T4. Regarding the proportion of the “Unassigned” category, the values from MiSeq, MinION T0 and MinION T4 were 11.9% (SD 11.7), 5.5% (SD 6.3) and 0.0% (SD 0.0), respectively. The values did not differ significantly for MinION T0 to MinION T4 (*p *value = 0.45 > 0.05), or MinION T0 to MiSeq (*p*- value = 0.36 > 0.05). Conversely, the MinION T4 value was significantly lower than that of MiSeq (*p *value = 0.044). At the genus level, the bacterial compositions from MinION T4 showed greater taxonomic resolution than those from MiSeq.

## Discussion

We investigated whether fractional changes in taxonomic assignment and bacterial community composition exist in the comparison of PCR conditions using the MinION sequencer (Oxford Nanopore Technologies) with mock community and environmental samples. We also compared the results from the bacterial community samples from MinION with those from the MiSeq sequencer (Illumina). The search for new analytical tools with shorter run times has progressed considerably with the third-generation MinION sequencing platform, because of its rapid and easy handling^37^. The increased information content inherent from longer read lengths help researchers with alignment-based taxonomy assignment^17^. With the ability to generate longer read lengths, MinION analysis can target the entire 16S rRNA gene coding region to offer highly accurate, sensitive and rapid pathogen detection^20,38^. Our goal was to determine the better conditions under which accurate bacterial community structuring data could be obtained using a nanopore sequencer. DNA amplification was performed for 35 cycles in all our PCR protocols. Several studies have shown that larger PCR cycle numbers cause chimera generation and interfere with bacterial community structure analysis^39,40^. Hence, minimizing the number of PCR cycles by optimizing the starting template conditions and concentrations is important^26^. However, in our situation, reducing the number of PCR cycles to less than 35 decreased the number of environmental DNA samples that were amplified. Tap water has relatively lower microbial density than that in other environmental samples such as sea water and soil^41^. Therefore, the procedures used in this study may also be applied to samples with low microbial cell densities, such as atmospheric (~ 10^4^ cells m^−3^)^42^ samples, too.

The sequencing data from the strictest filtration range (1,400–1,600 bases) provided 86% matching to *V. cholerae* in our database (Fig. 1). This indicates that high-resolution analysis at the species level is possible with MinION by eliminating extraneous read data. Jethro et al.^11^ stated that by using full-length sequences it is possible to classify nearly all environmental sequences into correct species. The read number (1,735 reads) after this treatment (43% of 3,994 reads in total) was used for subsequent analyses. Decreasing the read number caused no problem in this experiment because the precise mapping of only one species (*V. cholera*) was the main objective. Mitsuhashi et al.^23^ and Nakagawa et al.^43^ reported that a 5-min and 3-min running time on MinION, respectively, were enough for detecting specific bacteria. However, deeper sequencing is required to obtain better estimates of bacterial community structure and higher Goods coverage values^44,45^. We conducted a 48-h MinION operation for the mock communities and environmental samples to provide sufficient read numbers in our study (Table 2).

Optimal PCR conditions need to be established to obtain accurate bacterial community structure analyses using MinION. We therefore compared five different PCR conditions using mock communities in preliminary experiments (Table 1). The dissimilarity values within the communities were smaller with the T2 condition (longer annealing time than T0), the T3 condition (longer time for both annealing and extension than T0) and the T4 condition (longer time for all stages than T0), than that of the T0 condition (Fig. 2). These results suggest that the polymerase extension time does not affect the bacterial community structure analysis. Conversely, a shorter annealing time, as in the T3 condition, resulted in relatively higher dissimilarity values compared with those from the other cases (T2 and T4). Considering the higher dissimilarity achieved under T1 together with the results under T3, a longer annealing time was deemed necessary for the proper assessment of bacterial community structure using full-length 16S rRNA PCR analysis. As shown in Fig. 3, at the ATCC10, the difference between T0 and T4 condition is not significant. Whereas at the ATCC20, the bacterial composition obtained from T4 condition was closer to the theoretical values than those obtained from the T0 condition. This is more precise at the species-level (Supplemental Table S3). These results suggest that the T4 PCR conditions with longer reaction times provide better results than the T0 condition when the sample diversity is high. However, *Bifidobacterium* was not detected by MinION analysis using either the T0 or T4 conditions. Previous publications have shown that the universal primers commonly used for metagenomic analyses (such as the 27F primer) possess limitations related to amplification bias. The 27F forward primer used in the 16S Barcoding Kit (SQK-RAB204, Oxford Nanopore Technologies) contains three base-pair mismatches against *Bifidobacterium* (27F primer: 5′-AGAGTTTGATCMTGGCTCAG-3′); that is, the sequence of the *B. adolescentis* primer site is 5′-AGGGTTCGATTCTGGCTCA-3′ (the mismatched bases are underlined)^46^. Conversely, Hu et al*.*^47^, for example, detected *Bifidobacterium* species by sequencing with universal primers (384F and 806R) in MiSeq. Thus, primer sequence modifications are required to avoid preferential detection of particular taxa, so that a broad range of bacteria species is covered, as was the case here with our *B. adolescentis*.

MinION has lower read accuracy but can generate much longer read lengths than those from MiSeq. Nygaard et al*.*^48^ analysed building-dust microbiomes using MinION and MiSeq and showed that, at the genus and species levels, MinION reported greater taxonomic resolution than MiSeq. Long reads help alignment-based assignment of taxonomy as well, because of their increasing taxonomical information content. In this study, under the MinION T4 condition, all the environmental samples showed better taxonomic resolution at the genus level than that under MiSeq, the same as previously reported^17,48^. Many papers have been published on software developments and shorter running times with MinION. For example, Kai et al*.*^38^ reported on the possibility of decreasing the sequencing time of MinION by direct PCR approaches and found that a 3-min sequencing run generated a sufficient number of reads for taxonomic assignment and less than two hours was required for identifying appropriate bacterial species.

Characterization of environmental bacterial communities requires both qualitative and quantitative information through appropriate sequence read filtering as well as experimental procedures. Here, we have demonstrated, for the first time, that the accurate data on bacterial communities using MinION can be generated by comparing and choosing appropriate PCR conditions. The reaction condition in this study are the longest among PCR conditions compared to previous studies on bacterial community structure analysis with 16S rRNA with the MinION system (see Supplemental Table S4); however, using this condition we were able to obtain bacterial community structures that were comparable in quality with MiSeq.

## Methods

### Sample and DNA preparation

The full-length bacterial 16S rRNA gene from *V. cholerae* DNA, obtained through the courtesy of Dr. Taichiro Takemura (Nagasaki University, Japan), was used to examine the bioinformatics pipeline. PCR conditions evaluation was initially performed using a reference genomic DNA (Zymo Research Corp., Irvine, CA, USA; https://www.zymoresearch.com). The ZymoBIOMICS microbial community DNA standard (ZymoBIOMICS catalog # D6305) contains a mixture of genomic DNAs isolated from the pure cultures of eight bacterial and two fungal strains, and an equal molar quantity of 16S rDNA from each organism is provided. PCR conditions were examined for the mock community DNA samples (10 Strain Even Mix Genomic Material (MSA-1000) and 20 Strain Even Mix Genomic Material (MSA-1002); American Type Culture Collection (ATCC), Manassas, VA, USA) as well as environmental biofilm and water samples (that is, the insides of showerheads, bathtub inlets and showerhead feed water) in Japan. The biofilm samples were collected as described previously with a swab^49^. Two litter of showerhead feed water was filtered on-site using a 50-mL syringe (Terumo corporation, Tokyo, Japan) and 0.2-µm filter cartridge (Sterivex, Millipore, MA, USA). The samples were immediately put in a cool box, carried to the lab and kept at − 20 °C. DNA was extracted from these samples using the DNeasy PowerBiofilm Kit (QIAGEN, Germantown, MD, USA) with slight modification^50^. The extracted DNA was kept at − 20 °C after purification and precipitation using Dr. GenTLE precipitation carrier (Takara Bio Inc.). The concentrations and purities of the extracted DNA preparations were determined using the Spectro/Fluorometer (DS-11FX+, DeNovix, Wilmington, USA) and QuantiFluor dsDNA System (Promega, Madison, WI, USA).

### PCR conditions

Polymerase amplification efficiency was initially checked using 13 biofilm and water samples collected from bathrooms. Five different DNA polymerase enzymes were tested and MightyAmp DNA polymerase v. 2 (Takara Bio Inc.) provided the highest amplification efficiency among other polymerases used in our evaluation, as reported previously elsewhere^51^. The PCRs were conducted using a primer pair (27F and 1492R) specific for the 16S rRNA gene-targeting sequence contained in the library preparation kit (SQK-RAS201 or SQK-RAB204, Oxford Nanopore Technologies). Some samples were barcoded using a rapid amplicon barcoding kit (SQK-RAB201, Oxford Nanopore Technologies) according to the manufacturer’s protocol (see Supplemental Table S1 for details). The first PCR condition (T0) involved a pre-heating step at 98 °C for 2 min, 35 cycles at 98 °C for 10 s, 60 °C for 15 s and 68 °C for 2 min. Alternative PCR conditions (T1–T4) for the duration of each step are listed in Table 1. The PCR on *V. cholerae* DNA was performed using the T0 condition. Table 1 shows the four different PCR conditions (T1–T4) that were used with the ZymoBIOMICS mock community sample. The ATCC mock community and the environmentally-sourced samples were amplified using two different PCR conditions (T0 and T4). The amplified fragments were separated on 2% agarose gels, stained with Safelook Load-Green (Wako, Osaka, Japan), and visualized on the FAS Nano Gel Document System (Nippon Genetics, Tokyo, Japan).

### Nanopore sequencing library construction

After purifying the PCR products (50 μl each) with 30 μl of Agencourt AMPure XP beads (Beckman Coulter, Tokyo, Japan), the amount and purity of DNA eluted with 10 μl of buffer solution (10 mM Tris-HCl pH 8.0, with 50 mM NaCl) was determined using a Spectro/Fluorometer (DS-11FX+, DeNovix) and QuantiFluor dsDNA system (Promega). Purified amplicon DNA (100 or 50 fmol) was used as input DNA for the MinION-compatible libraries. The amplicons were added to 1 μl of rapid adapter (Oxford Nanopore Technologies) and incubated at room temperature for the required time.

### Nanopore sequencing and base-calling

**Figure 5:**
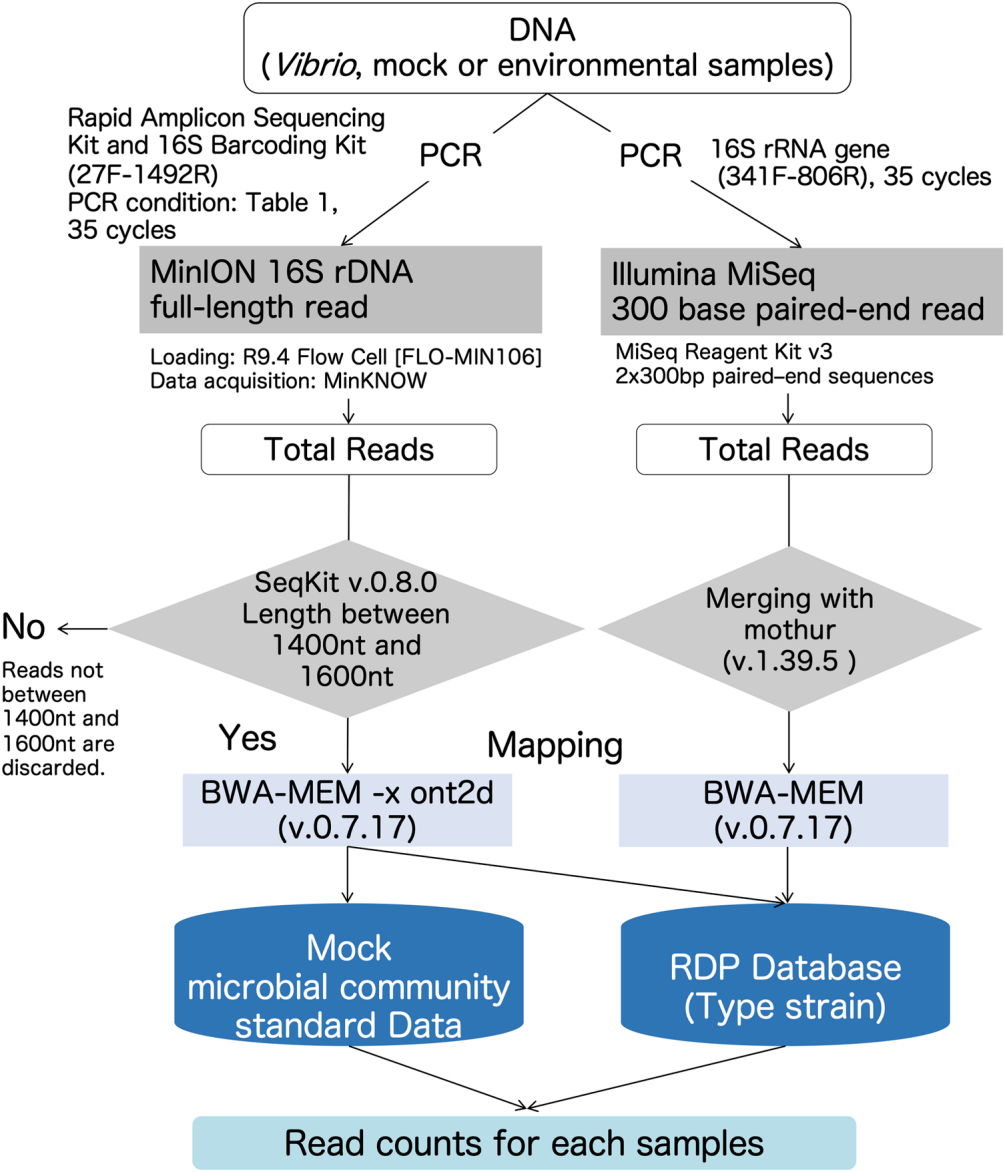
Overview of the experimental workflow. Experiments were performed as shown in the flowchart.

The nanopore sequencing libraries were separately run on FLO-MIN106 R9.4 flow cells (Oxford Nanopore Technologies) after performing platform quality control analysis. The amplicon library (11 μl) was diluted with running buffer (35 μl) containing 3.5 μl of nuclease-free water and 25.5 μl of loading beads. A 48-h sequencing protocol was initiated using the MinION control software, MinKNOW from v. 1.6.1-1.10.23 (Supplemental Table S1 contains the detailed information). MinION sequence reads (that is, fast5 data) were converted into fastq files using Albacore v. 1.2.1 or v. 2.1.3 software (Oxford Nanopore Technologies) and filtered with the threshold of a mean quality score over 7. (Supplemental Table S1 contains the detailed information). Figure 5 shows the study’s workflow.

### Nanopore sequencing data analysis

Sequence length distribution was examined for each base-called fastq file using FastQC (v 0.11. 2) (https://www.bioinformatics.babraham.ac.uk/projects/fastqc/)^52^. SeqKit 0.8.0 (https://bioinf.shenwei.me/seqkit/)^53^ and original ruby script were used to filter the sequence data by three lengths of 1,000–2,000, 1,200–1,800 and 1,400–1,600 to include the highest read length frequency (1,500 bases), with the filtering effects examined by calculating the hit ratio to *V. cholerae* in all the leads. All data except for *V. cholerae* were filtered with a read length of 1,400–1,600. After filtering, the sequence reads were mapped using BWA-MEM (v. 15. 0.7 or v. 0.7. 17; https://github.com/lh3/bwa)^33^ with the MinION analysis option (-x ont2d)^54^ to a database derived from the RDP (RDP Release 11, Update 5, Sept. 30. 2016; 3,356,809 aligned and annotated 16S rRNA sequences)^34^ and the top hit was used for the genus and species assignment. The RDP hierarchy browser (https://rdp.cme.msu.edu/hierarchy/hb_intro.jsp) was used with the following filters: strain = “Type”; source = “isolates”; size “≥ 1,200”; quality = “Good”; taxonomy = “Nomenclatural” to generate a downloaded set of 12,227 sequences.

### Illumina sequencing

The 16S rRNA sequencing library was constructed according to the Illumina 16S Metagenomic Sequencing Library Preparation protocol (Illumina) targeting the V3 and V4 hypervariable regions of the 16S rRNA genes using primers 341F (5′-CCTACGGGNGGCWGCAG-3′) and 805R (5′-GACTACHVGGGTATCTAATCC-3′)^16^. MightyAmp DNA Polymerase v. 2 (Takara Bio Inc.) was used for the PCRs. The initial PCR was performed using region-specific primers to ensure compatibility with the Illumina index and sequencing multiplex adapters. The amplified fragments were separated on 2% agarose gels, stained with Safelook Load-Green (Wako), and visualized on the FAS Nano Gel Document System (Nippon Genetics). The amount of purified DNA recovered was quantified using a Spectro/Fluorometer (DS-11FX+, DeNovix). An equimolar mixture of all PCR products was sent to a commercial company for 2 × 300 bp paired-end sequencing on the MiSeq platform using Illumina MiSeq v3 Reagent Kit (Fasmac, Kanagawa, Japan).

### Illumina sequencing data analysis

Illumina 16S rRNA amplicon sequence data were demultiplexed, and index sequences were removed using MiSeq Control Software (MCS) v2.6. Paired forward and reverse sequences were merged using ‘make.contings’ with the default parameter of mothur^32^ (v. 1.39.5). The merged sequence reads were assigned taxonomy using BWA-MEM^33^ against RDP^34^, using the same database and parameters without ‘ont2d’ option as our nanopore sequence data.

### Data analysis

All data analysis was carried out with R (v. 3.3.1)^55^. Bacterial community dissimilarities for the different PCR conditions were calculated by the Bray–Curtis index with the ‘vegan’ package (v. 2.5-5)^35^. Initially, MiSeq reads were randomly sampled to eliminate read number differences when comparing of unassigned percentages in the MiSeq, MinION T0 and MinION T4 runs. After normalization and confirming there were no significant differences between the number of reads (*p* value = 0.6758 > 0.05), the unassigned ratio was compared using Tukey’s honest significant difference test, and the variances in the data from the three groups were found not to be equal (*F* value < 0.01).

## Supplementary information

Supplementary Information.

## Data Availability

The mock microbial community DNA standards can be obtained from ZymoBIOMICS (catalog # D6305) and ATCC (catalog # MSA-1000 and MSA-1002). All the DNA sequences generated in the present study have been deposited in the DNA Data Bank of Japan (DDBJ) under the BioProject number PRJDB9684.
