# Supplementary material for: Evaluation of PCR conditions for characterizing bacterial communities with full-length 16S rRNA genes using a portable nanopore sequencer
Source: Sci Rep. 2020 Jul 28;10:12580. doi: 10.1038/s41598-020-69450-9 (PMC7387495; doi:10.1038/s41598-020-69450-9)
Supplement: Supplementary file 1 — Supplementary Information. [file 41598_2020_69450_MOESM1_ESM.pdf]

Supplementary material

Evaluation of PCR conditions for characterizing bacterial communities with full-length 16S rRNA genes using a portable nanopore sequencer

So Fujiyoshi[1,2]*, Ai Muto-Fujita[3], Fumito Maruyama[1,2]⁑

[1] Office of Industry-Academia-Government and Community Collaboration, Hiroshima University, 1-3-2 Kagamiyama, Higashi-Hiroshima, Hiroshima 739-8511, JAPAN

[2.] Laboratorio de Ecología Microbiana Aplicada, Departamento de Ciencias Químicas y Recursos Naturales, Scientific and Biotechnological Bioresource Nucleus (BIOREN-UFRO), Universidad de La Frontera, Ave. Francisco Salazar 01145, Temuco, CHILE

[3.] Data Science Center, Nara Institute of Science and Technology, Ikoma, Nara, JAPAN

Supplemental Table S1. Flow cell, MinKNOW and Albacore version information

Sample Name (PCR condition)	Flow cell version	MinKNOW version	Albacore version
Vibrio cholerae (T0 condition)	FLO-MIN 106 R9.4.1	ver. 1.6.11	ver. 1.2.1
ZymoBIOMICS mock community (T0 condition)	FLO-MIN 106 R9.4.1	ver. 1.6.11	ver. 1.2.1
B2 (T0 condition)	FLO-MIN 106 R9.4.1	ver. 1.6.11	ver. 1.2.1
B6 (T0 condition)	FLO-MIN 106 R9.4.1	ver. 1.7.3	ver. 1.2.1
B7 (T0 condition)	FLO-MIN 106 R9.4.1	ver. 1.7.3	ver. 1.2.1
B11 (T0 condition)	FLO-MIN 106 R9.4.1	ver. 1.7.3	ver. 1.2.1
B13 (T0 condition)	FLO-MIN 106 R9.4.1	ver. 1.7.3	ver. 1.2.1
B14 (T0 condition)	FLO-MIN 106 R9.4.1	ver. 1.7.3	ver. 1.2.1
ATCC_mock10 (T0 condition)	FLO-MIN 106 R9.4.1	ver. 1.7.14	ver. 1.2.1
ATCC_mock20 (T0 condition)	FLO-MIN 106 R9.4.1	ver. 1.7.14	ver. 1.2.1
ZymoBIOMICS mock community (T0, T1, T2, T3, T4 condition)	FLO-MIN 106 R9.4.1	ver. 1.10.23	ver. 2.1.3
B2, 6 (T4 condition)	FLO-MIN 106 R9.4.1	ver. 1.7.3	ver. 2.1.3
B7,11,13,14, ATCC10, ATCC20 (T4 condition)	FLO-MIN 106 R9.4.1	ver. 1.7.3	ver. 2.1.3

Supplemental Table S2. The proportion of sequences identified at the genus and species level of each PCR condition

Mock community (ZymoBIOMICS)	MinION PCR conditions									
	T0		T1		T2		T3		T4	
	Genus	Species	Genus	Species	Genus	Species	Genus	Species	Genus	Species
Pseudomonas aeruginosa	1.6%	1.3%	5.9%	3.5%	4.1%	2.1%	4.1%	1.9%	4.4%	2.3%
Escherichia/Shigella coli	4.4%	0.9%	11.7%	2.1%	5.8%	1.0%	4.6%	1.0%	4.4%	0.9%
Enterococcus faecalis	8.2%	3.1%	4.9%	2.5%	8.8%	4.1%	9.1%	3.7%	8.5%	3.6%
Salmonella enterica	10.7%	10.0%	25.2%	24.0%	12.1%	11.4%	10.6%	9.8%	9.6%	9.0%
Staphylococcus aureus	15.7%	3.5%	7.0%	1.8%	14.9%	3.6%	15.3%	3.7%	16.5%	4.0%
Bacillus subtills	22.8%	5.8%	11.6%	3.3%	19.5%	5.2%	21.2%	5.2%	19.9%	5.1%
Listeria monocytogenes	10.5%	1.7%	6.1%	0.7%	10.8%	1.4%	10.8%	1.7%	11.0%	1.6%
Lactobacillus fermentum	5.9%	3.2%	4.4%	2.9%	6.8%	4.4%	7.1%	4.3%	8.9%	5.5%
Others	18.9%	70.5%	23.2%	59.3%	17.4%	66.8%	18.3%	68.7%	16.9%	68.1%
Dissimilarity values	0.284	0.705	0.400	0.722	0.238	0.672	0.247	0.687	0.243	0.680

Supplemental Table S3. The proportion of sequences identified at the genus and species level of each sample and condition

Mock community (ATCC10)	MiSeq		MinION_PCR(T0)		MinION_PCR(T4)	
	Genus	Species	Genus	Species	Genus	Species
Bacillus cereus	12.2%	0.5%	3.3%	2.7%	3.7%	2.9%
Bifidobacterium adolescentis	7.6%	5.2%	ND	ND	ND	ND
Clostridium beijerinckii	20.7%	5.7%	16.1%	12.5%	16.2%	12.9%
Deinococcus radiodurans	8.5%	8.1%	13.4%	13.3%	13.8%	13.7%
Enterococcus faecelis	4.3%	3.3%	6.6%	6.1%	5.2%	4.9%
Escherichia coli	9.8%	0.1%	14.1%	0.4%	11.7%	0.5%
Lactobacillus gasseri	8.1%	6.7%	8.3%	7.7%	6.6%	6.1%
Rhodobacter sphaeroides	3.3%	1.5%	7.0%	3.8%	9.7%	7.1%
Staphylococcus epidermidis	10.5%	5.9%	9.5%	2.8%	11.2%	2.2%
Streptococcus mutans	10.8%	9.7%	11.2%	10.9%	9.1%	9.0%
Others	4.1%	53.3%	10.5%	39.8%	12.8%	40.8%
Dissimilarity values	0.184	0.533	0.253	0.465	0.257	0.473

ND: Not Detected

Mock community (ATCC20)	MiSeq		MinION_PCR(T0)		MinION_PCR(T4)	
	Genus	Species	Genus	Species	Genus	Species
Acinetobacter baumannii	8.2%	7.0%	4.4%	1.7%	6.9%	6.5%
Actinomyces odontolyticus	1.7%	1.6%	0.1%	ND	0.1%	0.1%
Bacillus cereus	5.2%	0.3%	2.0%	0.4%	2.6%	1.8%
Bacteroides vulgatus	9.3%	9.2%	1.2%	1.0%	4.8%	4.8%
Bifidobacterium adolescentis	4.1%	2.6%	ND	ND	ND	ND
Clostridium beijerinckii	12.2%	3.5%	8.9%	3.0%	8.4%	6.8%
Deinococcus radiodurans	3.5%	3.2%	6.8%	5.3%	7.4%	7.3%
Enterococcus faecalis	2.4%	1.8%	4.4%	1.8%	2.7%	2.6%
Escherichia coli	4.4%	0.2%	6.9%	1.4%	9.4%	0.4%
Helicobacter pylori	3.8%	3.3%	5.9%	4.4%	6.0%	5.9%

Lactobacillus gasseri	4.2%	3.3%	6.3%	2.4%	3.7%	3.3%
Neisseria meningitidis	5.8%	4.6%	2.6%	1.4%	5.5%	0.3%
Porphyromonas gingivalis	7.8%	7.7%	0.8%	0.7%	2.8%	2.7%
Propionibacterium acnes	0.9%	0.9%	0.5%	0.4%	0.7%	0.7%
Pseudomonas aeruginosa	2.9%	2.3%	2.1%	1.1%	4.3%	4.0%
Rhodobacter sphaeroides	0.9%	0.4%	3.0%	1.2%	5.0%	3.0%
Staphylococcus aureus	9.6%	1.6%	10.2%	1.1%	11.9%	0.6%
Staphylococcus epidermidis		3.0%		1.8%		1.3%
Streptococcus agalactiae	10.7%	4.7%	15.3%	4.5%	11.1%	5.6%
Streptococcus mutans		5.0%		4.1%		5.2%
Others	2.3%	33.8%	18.7%	62.2%	6.7%	37.1%
Dissimilarity values	0.216	0.427	0.338	0.625	0.233	0.444

ND: Not Detected

Environmental sample (B2)	MiSeq	MinION_PCR(T0)	MinION_PCR(T4)
Methylobacterium	26.2%	40.1%	39.2%
Moraxella	8.0%	9.6%	11.9%
Sphingomonas	32.0%	43.0%	42.8%
Enhydrobacter	6.1%	1.7%	1.7%
Chryseobacterium	2.2%	0.2%	0.3%
Brevundimonas	1.4%	0.6%	1.2%
Sphingobium	1.4%	1.8%	1.4%
Asticcacaulis	0.3%	0.0%	0.0%
Rhizobium	0.1%	0.1%	0.1%
Acidovorax	0.3%	0.4%	0.1%
Burkholderia	0.0%	0.0%	0.0%
Acinetobacter	0.1%	0.1%	0.1%
Novosphingobium	0.1%	0.1%	0.1%
Streptococcus	0.0%	0.0%	0.0%
Unassigned	21.3%	2.0%	0.0%
Others	0.5%	0.3%	1.1%

Environmental sample (B6)	MiSeq	MinION_PCR(T0)	MinION_PCR(T4)
Methylobacterium	71.7%	62.3%	71.6%

Blastomonas	12.8%	13.5%	13.2%
Sphingomonas	9.8%	6.9%	8.5%
Acinetobacter	3.5%	2.7%	2.4%
Bdellovibrio	0.2%	0.0%	0.0%
Sphingopyxis	0.2%	0.2%	0.4%
Desulfovibrio	0.2%	0.0%	0.0%
Runella	0.1%	0.0%	0.0%
Polymorphobacter	0.1%	0.1%	0.0%
Chryseolinea	0.1%	0.0%	0.0%
Caulobacter	0.1%	0.1%	0.2%
Pseudomonas	0.1%	0.0%	0.1%
Desulfarculus	0.1%	0.0%	0.0%
Emticicia	0.1%	0.0%	0.0%
Unassigned	0.1%	12.1%	0.0%
Others	0.9%	2.2%	3.5%

Environmental sample (B7)	MiSeq	MinION_PCR(T0)	MinION_PCR(T4)
Roseomonas	19.3%	31.0%	37.7%
Actinomycetospora	17.5%	3.2%	1.9%
Geminicoccus	4.5%	2.1%	2.8%
Sphaerobacter	3.4%	0.0%	0.1%
Luteimonas	2.2%	6.2%	5.0%
Pelotomaculum	2.1%	0.0%	0.0%
Sphingomonas	3.1%	2.7%	3.9%
Oscillochloris	1.6%	0.1%	0.1%
Fibrisoma	1.2%	0.4%	0.4%
Brevundimonas	1.9%	1.2%	2.6%
Kocuria	1.2%	0.7%	0.5%
Methylobacterium	4.6%	5.5%	6.2%
Salinactinospora	0.7%	0.0%	0.0%
Dehalogenimonas	0.8%	0.0%	0.0%
Unassigned	0.1%	15.2%	0.0%
Others	35.7%	31.9%	38.8%

Environmental sample (B11)	MiSeq	MinION_PCR(T0)	MinION_PCR(T4)
Deinococcus	34.4%	6.8%	10.4%
Moraxella	12.8%	50.6%	44.3%

Enhydrobacter	7.1%	8.6%	1.9%
Barrientosiimonas	3.9%	3.7%	3.7%
Chryseobacterium	2.5%	0.3%	3.7%
Sphaerobacter	1.5%	0.0%	0.2%
Nakamurella	2.5%	2.5%	2.8%
Brevibacterium	1.6%	1.2%	1.9%
Sphingomonas	3.6%	6.4%	8.9%
Niabella	1.2%	1.8%	2.7%
Actinomycetospora	0.8%	0.3%	0.3%
Brevundimonas	0.9%	0.7%	1.9%
Streptococcus	1.1%	0.2%	0.3%
Methylobacterium	0.9%	1.1%	2.1%
Unassigned	18.2%	1.5%	0.0%
Others	7.1%	14.3%	14.8%

Environmental sample (B13)	MiSeq	MinION_PCR(T0)	MinION_PCR(T4)
Blastocatella	9.4%	3.3%	2.2%
Actinomycetospora	14.0%	12.3%	8.9%
Methylobacterium	12.4%	23.7%	25.8%
Longilinea	4.2%	0.2%	0.1%
Belnapia	2.3%	1.0%	2.1%
Oscillochloris	1.4%	0.1%	0.2%
Exiguobacterium	1.4%	0.0%	0.0%
Sphingorhabdus	1.2%	0.0%	0.0%
Pyrinomonas	1.1%	0.0%	0.2%
Caloribacterium	1.1%	0.0%	0.0%
Sphingomonas	4.2%	4.7%	6.6%
Pseudonocardia	2.5%	1.9%	1.4%
Kribbella	2.5%	1.6%	1.3%
Roseomonas	1.3%	2.4%	3.7%
Unassigned	4.4%	1.5%	0.0%
Others	36.7%	47.1%	47.6%

Environmental sample (B14)	MiSeq	MinION_PCR(T0)	MinION_PCR(T4)
Deinococcus	33.7%	9.6%	7.6%
Moraxella	11.4%	50.9%	50.3%
Enhydrobacter	6.8%	7.8%	2.7%
Chryseobacterium	2.9%	0.4%	2.0%

Barrientosiimonas	2.8%	3.8%	2.7%
Sphaerobacter	1.8%	0.0%	0.1%
Brevibacterium	1.6%	1.5%	1.0%
Niabella	1.3%	1.5%	2.3%
Nakamurella	2.1%	2.7%	2.1%
Sphingomonas	3.1%	7.0%	11.6%
Actinomycetospora	0.9%	0.3%	0.2%
Bosea	0.5%	1.5%	2.2%
Sphingobium	0.5%	0.6%	1.1%
Brevundimonas	0.4%	0.6%	2.2%
Unassigned	25.5%	0.5%	0.0%
Others	4.8%	11.3%	11.9%

Supplemental Table S4. PCR details for full-length 16S rRNA analyses using MinION

Ref	This study (PCR condition T4)	Nanopore 16S Barcoding Kit protocol (ver. RAB_9053_v1_revL_14Aug2019)	Shin et al. SciRep 6:29681 (2016)	Benítez-Páez et al. GigaScience 5, 4 (2016)	Mitsuhashi et al. Sci Rep. 7:5657 (2017)	Kai et al. FEBS Open Bio. 9:548-557 (2019)
Ref No.	–	https://community.nanoporetech.com/protocols/16S-barcoding-sequencing/checklist_example.pdf	[56]	[22]	[23]	[38]
Enzyme	MightyAmp DNA Polymerase Ver. 2 (Takara Bio, Inc., Tokyo, Japan)	LongAmp Taq 2× Master Mix (New England Biolabs, MA, USA)	Phusion High-Fidelity polymerase (Thermo Scientific, MA, USA)	Phusion High-Fidelity polymerase (Thermo Scientific, MA, USA)	KAPA HiFi HotStart ReadyMix (KAPA Biosystems, MA, USA)	LongAmp Taq 2× Master Mix (New England Biolabs, MA, USA)
Denaturation	95°C for 1 min	95°C for 20 sec	98°C for 10 sec	95°C for 20 sec	98°C for 20 sec	95°C for 20 sec
Annealing	60°C for 1 min	55°C for 30 sec	47°C for 30 sec	47°C for 30 sec	60°C for 15 sec	55°C for 30 sec
Extension	68°C for 3 min	65°C for 2 min	72°C for 1 min	72°C for 1 mi	72°C for 15 sec	65°C for 2 min
No. of cycles	35	25	15	30	25	25

Reference

56. Shin, J. et al. Analysis of the mouse gut microbiome using full-length 16S rRNA amplicon sequencing. Sci. Rep. 6, 29681 (2016).
